# Effect of Eicosapentaenoic Acid and Docosahexaenoic Acid on Myogenesis and Mitochondrial Biosynthesis during Murine Skeletal Muscle Cell Differentiation

**DOI:** 10.3389/fnut.2018.00015

**Published:** 2018-03-12

**Authors:** Tun-Yun Hsueh, Jamie I. Baum, Yan Huang

**Affiliations:** ^1^Department of Animal Science, Division of Agriculture, University of Arkansas, Fayetteville, AR, United States; ^2^Department of Food Science, Division of Agriculture, University of Arkansas, Fayetteville, AR, United States

**Keywords:** eicosapentaenoic acid and docosahexaenoic acid, mitochondria, maternal nutrition, C2C12 myoblasts, muscle growth and development

## Abstract

Polyunsaturated fatty acids are important nutrients for human health, especially omega-3 fatty acids such as eicosapentaenoic acid (EPA) and docosahexaenoic acid (DHA), which have been found to play positive roles in the prevention of various diseases. However, previous studies have reported that excessive omega-3 fatty acids supplement during pregnancy caused side effects such as slower neural transmission times and postnatal growth restriction. In this study, we investigated the effect of EPA and DHA on mitochondrial function and gene expression in C2C12 myoblasts during skeletal muscle differentiation. C2C12 myoblasts were cultured to confluency and then treated with differentiation medium that contained fatty acids (50-µM EPA and DHA). After 72 h of myogenic differentiation, mRNA was collected, and gene expression was analyzed by real-time PCR. Microscopy was used to examine cell morphology following treatment with fatty acids. The effect of EPA and DHA on cellular oxygen consumption was measured using a Seahorse XF24 Analyzer. Cells treated with fatty acids had fewer myotubes formed (*P* ≤ 0.05) compared with control cells. The expression of the genes related to myogenesis was significantly lower (*P* ≤ 0.05) in cells treated with fatty acids, compared with control cells. Genes associated with adipogenesis had higher (*P* ≤ 0.05) expression after treatment with fatty acids. Also, the mitochondrial biogenesis decreased with lower (*P* ≤ 0.05) gene expression and lower (*P* ≤ 0.05) mtDNA/nDNA ratio in cells treated with fatty acids compared with control cells. However, the expression of genes related to peroxisome biosynthesis was higher (*P* ≤ 0.05) in cells treated with fatty acids. Moreover, fatty-acid treatment reduced (*P* ≤ 0.05) oxygen consumption rate under oligomycin-inhibited (reflecting proton leak) and uncoupled conditions. Our data imply that fatty acids might reduce myogenesis and increase adipogenesis in myotube formation. Fatty acids may also decrease cell metabolism by reducing mitochondrial biogenesis as well as respiration rate. This study suggests that the maternal overdosage of EPA and DHA may influence fetal muscle development, increase intramuscular adipose tissue deposition in offspring, and have a long-term effect on the development of metabolic diseases such as obesity and diabetes in adult offspring.

## Introduction

The polyunsaturated fatty acids (PUFAs), especially *n* − 3 fatty acids such as eicosapentaenoic acid (EPA, 20:5, *n* − 3) and docosahexaenoic acid (DHA, 22:6, *n* − 3), mainly derived from fish oil and seaweed, phytoplankton, and camelina (1), have been found to play positive roles in human health (2). A series of studies have shown the efficient function of EPA and DHA in preventing body overweight and obesity (3–6). *n* − 3 fatty acids are widely considered as a dietary supplement during pregnancy, because of the benefit to fetal neurodevelopment and infant birth weight (7). Although, overdosage of EPA and DHA is rare, it was reported that adverse effects such as the risk of bruising or bleeding caused by high intake of EPA and DHA in extreme circumstances (anticoagulants and antiplatelets treatments) were observed in several controlled human intervention studies (8). Clinical research showed that EPA and DHA supplementation during pregnancy accumulates in fetal tissues and causes a longer gestation (9). However, no study has addressed the question whether high intake of EPA and DHA affects fetal muscle development during gestation.

Skeletal muscle composes 40–50% or body weight and is an essential organ for glucose and fatty-acid utilization (10). Fetal stage is crucial for skeletal muscle development because of the limited increase in muscle fiber number after birth (11). During fetal muscle development, mesenchymal stem cells undergo myogenesis, adipogenesis, and fibrogenesis. The muscle growth in the postnatal stages occurs mainly due to the increase of the diameter and length of muscle fibers instead of muscle fiber (muscle cell) numbers. This indicates that muscle stem cells can be utilized as an *in vitro* model to study the muscle growth and development during the fetal stage. Comparing with central neuro system and cardiovascular tissue, skeletal muscle has a relatively lower priority of nutrition repartitioning which makes the development of skeletal muscle especially vulnerable to nutritional change (12). Studies in a sheep model indicated that maternal obesity induced by high-energy diet showed downregulation effect on fetal myogenesis and impaired skeletal muscle development in offspring, but increased number and size of intramuscular adipocytes in the fetus at mid- and late-gestation (13–16). Due to the metabolic importance, fetal skeletal muscle is considered as one of the target organs of lipid accumulation that induced by a high-fat or high-carbohydrate maternal diet (17, 18). It raises up a question whether a high-energy maternal diet rich in PUFAs similarly affects fetal muscle cell differentiation and adipose deposition during gestation.

In most eukaryotes, mitochondria are primary organelles that response for energy metabolism which derived from the breakdown of carbohydrates and fatty acids. It was reported that the *n* − 3 PUFAs could cause higher oxidation levels of mitochondrial fatty acids in the myocardium (19–22). EPA and DHA individually increase mitochondrial biosynthesis in skeletal muscle stem cells (23–25). Therefore, we hypothesize that EPA and DHA treatment on myofibroblasts (C2C12 cells) may affect the myogenesis and adipogenesis *via* metabolic changes in mitochondria.

The objective of the current study was to measure the effect of maternal EPA and DHA supplement on myotube formation and potential pathways related to adipose deposition and mitochondrial biosynthesis using myofibroblasts as *in vitro* model.

## Materials and Methods

### Cell Culture

Murine C2C12 myoblasts (cells were kindly donated by Dr. Jamie Baum from University of Arkansas) were cultured in Dulbecco’s modified Eagle’s medium (DMEM) with 10% fetal bovine serum and 1U penicillin-streptomycin at 37°C in a 5% CO2 atmosphere. When cells confluency reached more than 90%, control group (CON) was induced to fuse and form myotubes by switching to the differentiation medium (DM) containing 2% horse serum and 1U penicillin-streptomycin, while 50-µM EPA and 50-µM DHA were added to DM in the fatty-acid treatment group (FA). DM with or without fatty acids was changed every 24 h for 3 days.

### Hematoxylin and Eosin (H&E) Staining

Myotube formation is the direct observation of myogenesis. Myotube formation was observed by conducting H&E staining as described previously (26). Briefly, cells in 6-well plates (three for CON group and three for FA group) were fixed with ice-cold 100% ethanol, then froze in −20°C for 7 min. Hematoxylin stain was added to each well and stained for 5 min. Cells were rinsed with running tap water. Eosin stain was added in wells and stained for 30 s. Cells were rinsed with running tap water and rinse with phosphate-buffered saline. Cells were dried at room temperature before coverslips were mounted on the cells. Pictures were taken under a microscope (Nikon Eclipse Ts2R, Nikon, Melville, NY, USA) with the camera (Nikon DS-Fi3 digital camera system) and software (NIS-Elements BR, Nikon, Melville, NY, USA). Three pictures were randomly taken from each well, and total nine pictures for each group were analyzed for the myotube formation. The diameter and number of myotubes were measured using ImageJ software (US National Institutes of Health).

### Real-Time PCR

Gene expression related to myogenesis, adipogenesis, mitochondrial biosynthesis, and peroxisome biosynthesis were measured by quantitative real-time PCR. Total RNA was extracted from cells with the TRIzol reagent (Fisher, Pittsburgh, PA, USA). The concentration of total RNA was assessed by Nanodrop OneC (Thermo Scientific, Waltham, MA, USA), and quality was examined in the absorption ratio of OD_260 nm_/OD_280 nm_. The cDNA was synthesized from the RNA with iScript cDNA synthesis kit (Bio-Rad, Richmond, CA, USA). Real-time PCR was carried out by using SYBR Green Supermix (Bio-Rad, Richmond, CA, USA) on CFX Connect Real-Time PCR Detection System (Bio-Rad, Richmond, CA, USA). The oligonucleotide primers (Table 1) used were designed with NCBI database and Primer Quest (IDT.com). Each reaction yielded amplicons from 80 to 200 bp. PCR conditions were as follows: 30 s at 95°C, 30 s at 55°C, and 40 s at 72°C for 40 cycles. After amplification, a melting curve (0.01°C/s) was used to confirm product purity, and the PCR products were electrophoresed to confirm the targeted sizes. Results are expressed relative to β-actin. Data were analyzed using the ΔΔCT method and β-actin gene was the reference gene.

**Table 1:** List of primers.

Primers	Accession no.	Forward sequence	Reverse sequence
MyoD	NM_010866	TCTGGAGCCCTCCTGGCACC	CGGGAAGGGGGAGAGTGGGG
MyoG	NM_031189	GCAATGCACTGGAGTTCG	ACGATGGACGTAAGGGAGTG
MRF4	NM_008657	GTGGACCCCTACAGCTACAAACC	TGGAAGAAAGGCGCTGAAGAC
Pax7	NM_011039	CTCAGTGAGTTCGATTAGCCG	AGACGGTTCCCTTTGTCGC
PMP70	NM_008991	AAGAATGGCGATGGCAAGACT	TGTGAAACGGTAAAGAGGGTGAT
Pex2	NM_008994	TGAAGGAACCACTTAGAAATTACAGA	CCAGGGCCTTATTCAGTTCA
Pex19	NM_023041	CAGAGTGAGATGTGTTAGGAGATG	GTGCCAAGGAGACGAAGAC
ERRa	NM_007953	GGTGTGGCATCCTGTGAGGC	AGGCACTTGGTGAAGCGGCA
TFAM	NM_009360	GCTTGGAAAACCAAAAAGAC	CCCAAGACTTCATTTCATT
PGC1a	NM_008904	TCCTCTGACCCCAGAGTCAC	CTTGGTTGGCTTTATGAGGAGG
aP2	NM_024406	CGACAGGAAGGTGAAGAGCATCATA	CATAAACTCTTGTGGAAGTCACGCCT
C/EBPa	NM_007678	GCAAGCCAGGACTAGGAGAT	AATACTAGTACTGCCGGGCC
C/EBPb	NM_001287738	AAGCTGAGCGACGAGTACAAGATG	TTGAACAAGTTCCGCAGGGTGCT
PPARg	NM_001127330	GATGTCTCACAATGCCATCAG	TCAGCAGACTCTGGGTTCAG
CPT1	NM_009948	TATAACAGGTGGTTTGACA	CAGAGGTGCCCAATGATG
FAT	NM_001159558	CTCCTAGTAGGCGTGGGTCT	CACGGGGTCTCAACCATTCA
Rpl7	NM_011291	GAAGCTCATCTATGAGAAGGC	AAGACGAAGGAGCTGCAGAAC
β-actin	NM_007393	AAATCGTGCGTGACATCAAA	AAGGAAGGCTGGAAAAGAGC
mtDNA(Ttll3)	NM_133923	CGATAAACCCCGCTCTACCT	AGCCCATTTCTTCCCATTTC
nDNA (27)		CCTTGGGTCCTTGGCTTCGTTCCT	CTCAGCAATCAGCCGTCCAATTCCTA

### Oxygen Consumption Measurement

Mitochondrial activities and metabolic levels can be determined by conducting oxygen consumption measurement. Oxygen consumption rate (OCR) of C2C12 cells was measured in real time using a Seahorse XF24 extracellular flux analyzer (Seahorse Bioscience, North Billerica, MA, USA) (28). C2C12 cells were seeded at 3 × 10^4^ per well in 24-well XF plates. DM with or without EPA and DHA was then used to culture cells for 72 h once cells reached confluence. The cells were refreshed with non-buffered pH7.4 medium (25-mM glucose) and incubated for 1 h in a non-CO2 incubator. A Seahorse XF24 Analyzer was then used to measure the OCR.

### Statistical Analyses

Differences between groups were assessed for significance by the unpaired Student’s *t*-test with the assumption of equal variances, and arithmetic means ± SEM are reported. Statistical significance was considered as *P* ≤ 0.05.

## Results

### Myotube Formation in CON and FA Groups

**Figure 1:**
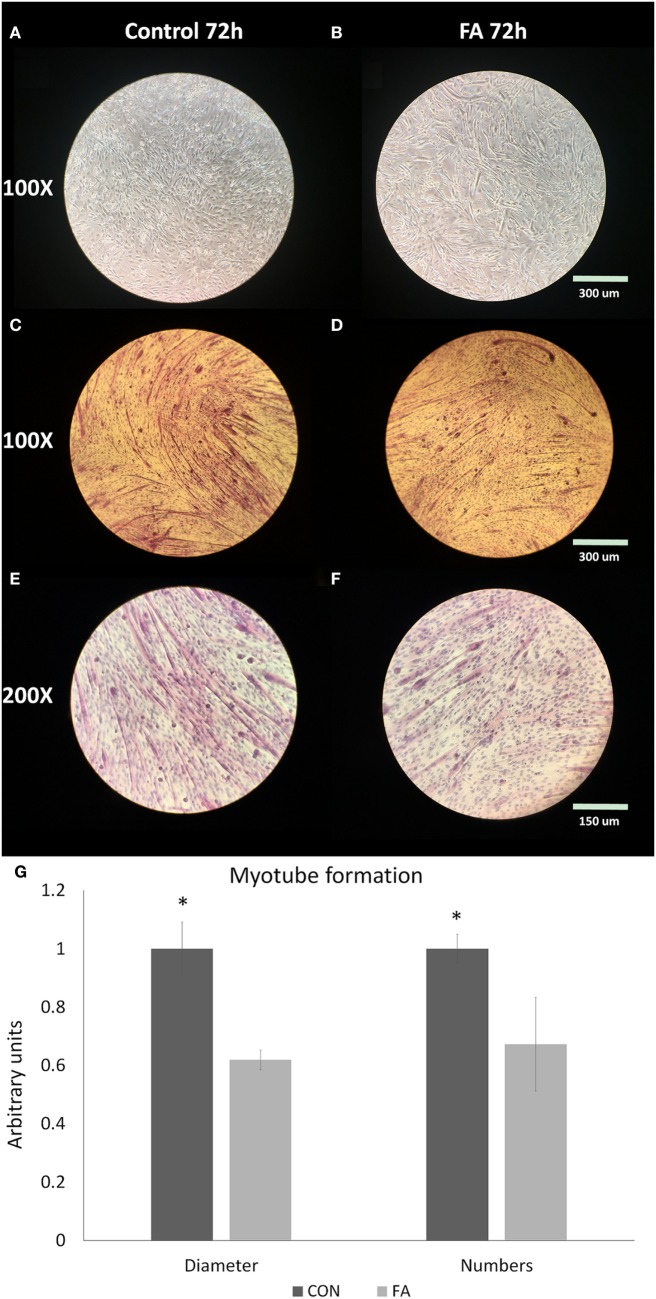
Myotube formation of C2C12 cells in control (CON) and EPA/DHA (FA) medium. **(A–F)** H&E staining of C2C12 cells. Pictures are shown in different magnification: 100× and 200×. **(G)** The diameter and length of myotubes measured based on the H&E staining images. Data show the change of the diameter and numbers of myotubes in FA group compared with CON group. The relative changes were calculated in arbitrary units. *P < 0.05; *n* = 9 pictures for each group.

After induced differentiation, rod-shaped C2C12 myoblasts fused and elongated to develop into nascent myotubes. Fewer myotubes with smaller diameter were observed histochemically in FA group than in CON group (Figures 1A–F). The average diameter of myotubes in FA group was 38.10 ± 3.33% lower (*P* < 0.05) than in CON group. The total myotube number in the field in FA group was 32.73 ± 16.07% lower (*P* < 0.05) than in CON group (Figure 1G).

### mRNA Expression of Genes Affected by FA Treatment

**Figure 2:**
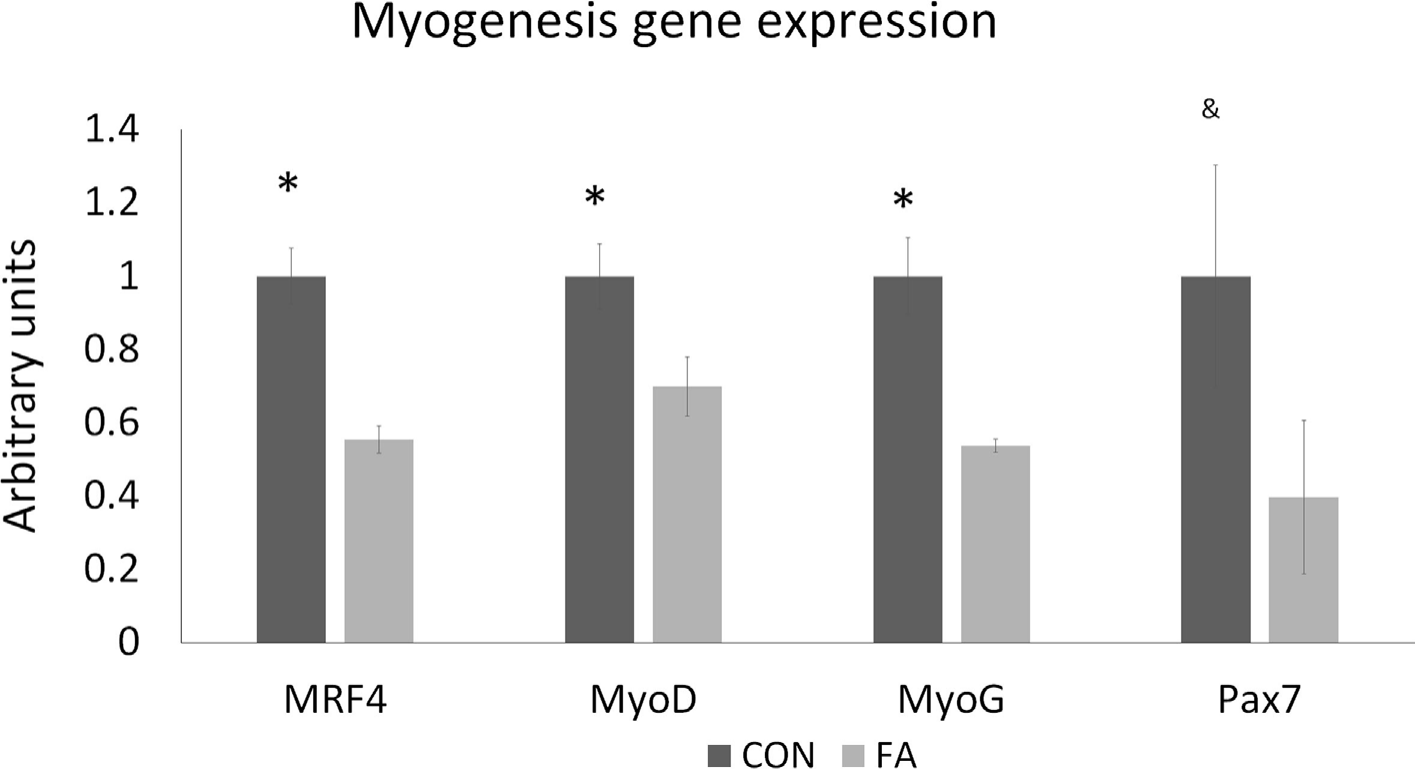
The relative gene expression related to myogenesis of C2C12 cells in control (CON) and EPA/DHA (FA) medium. Data show the change of their mRNA expression in FA group compared with CON group. The relative expressions were calculated in arbitrary units. *P < 0.05 and ^&^ *P* < 0.1; *n* = 3.

To determine the effect of EPA and DHA media supplement on the growth of the cells, we conducted qPCR targeted on the genes related to myogenesis, adipogenesis, mitochondrial biogenesis, and peroxisome biogenesis. Most of the myogenesis-related genes were downregulated by FA treatment (Figure 2). The expressions of MRF4, MyoD, and MyoG were all decreased (44.61 ± 3.66, 30.12 ± 8.07, and 46.25 ± 1.83%, respectively, *P* < 0.05) in FA treatment group compared with CON group. Pax7 was tended to be suppressed by FA treatment (60.31 ± 20.93%, *P* = 0.09) compared with CON group.

**Figure 3:**
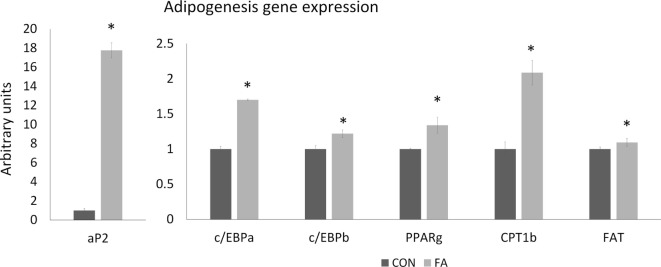
The relative gene expression related to adipogenesis of C2C12 cells in control (CON) and EPA/DHA (FA) medium. Data show the change of their mRNA expression in FA group compared with CON group. The relative expressions were calculated in arbitrary units. *P < 0.05; *n* = 3.

The mRNA expression of genes related to adipogenesis in C2C12 cells was increased in FA group (Figure 3). aP2 expression in FA group is 17.76 ± 0.80 times (*P* < 0.05) of the expression in CON group. The expressions of c/EBPα, c/EBPb, PPARγ, CPT1b, and FAT were all higher (70.08 ± 1.04, 21.84 ± 5.35, 34.06 ± 11.33, 108.82 ± 17.47, and 9.40 ± 5.42%, respectively, *P* < 0.05) in FA treatment group than in CON group.

**Figure 4:**
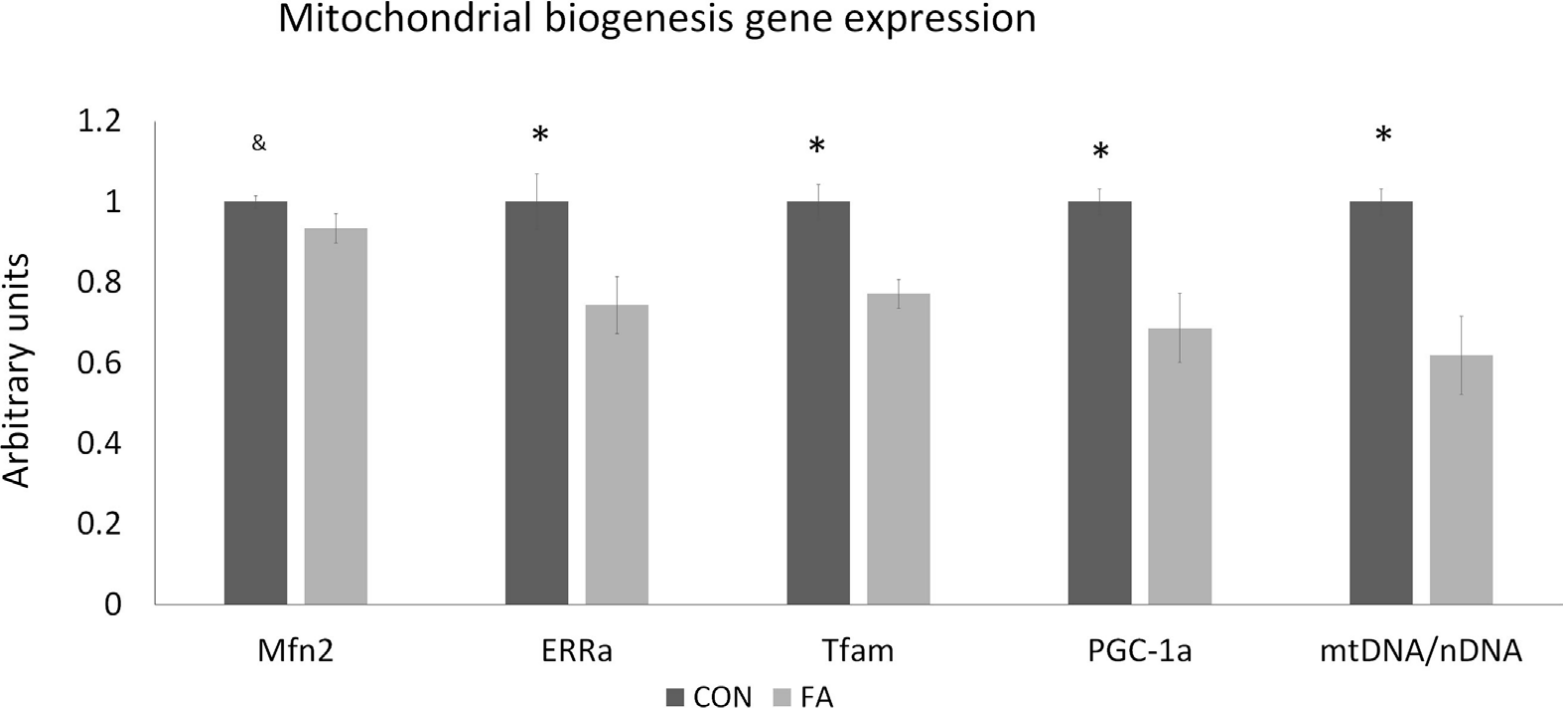
The relative gene expression related to mitochondrial biogenesis and function of C2C12 cells in control (CON) and EPA/DHA (FA) medium. Data show the change of their mRNA expression in FA group compared with CON group. The relative expressions were calculated in arbitrary units. *P < 0.05 and ^&^ *P* < 0.1; *n* = 3.

FA supplement suppressed gene related to mitochondrial function and biogenesis (Figure 4). The mRNA expression of ERRα (estrogen-related receptor alpha), TFAM (mitochondrial transcription factor A), and PGC1α (peroxisome proliferator-activated receptor gamma coactivator 1 alpha) were downregulated (25.63 ± 7.10, 22.87 ± 3.64, and 31.33 ± 8.62%, respectively, *P* < 0.05) by FA compared with CON group. Mfn2 (mitofusin 2) was tended to be decreased (6.61 ± 3.64%, *P* = 0.09) in FA group compared with CON group. The ratio of mtDNA to nDNA was significantly lower (38.07 ± 9.68%, *P* < 0.05) in FA-treated cells than in the CON cells.

**Figure 5:**
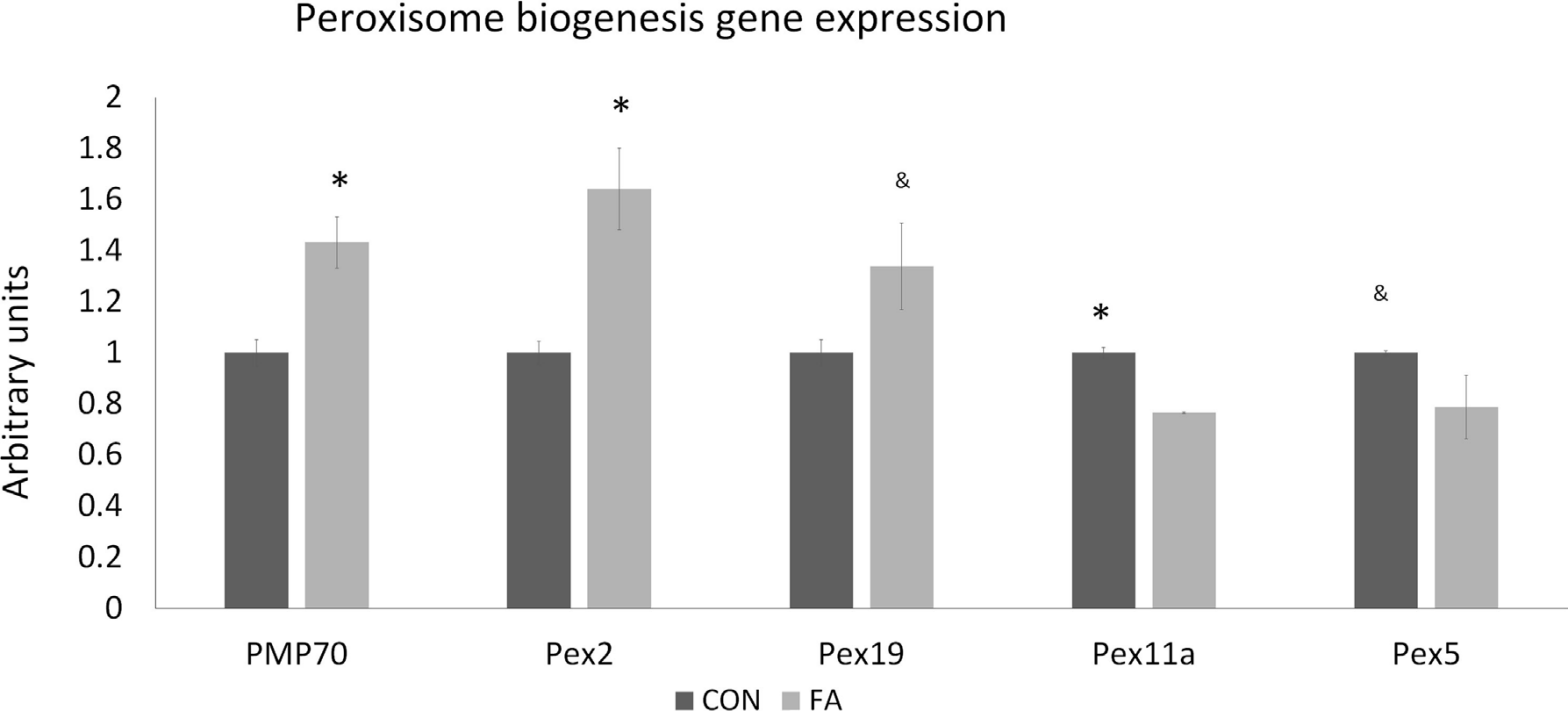
The mRNA expression of the genes related to peroxisome biogenesis of C2C12 cells in control (CON) and EPA/DHA (FA) medium. Data show the change of their mRNA expression in FA group compared with CON group. The relative expressions were calculated in arbitrary units. *P < 0.05 and ^&^ *P* < 0.1; *n* = 3.

Genes related to peroxisome biogenesis are also affected by FA treatment (Figure 5). PMP70 and Pex2 were significantly upregulated (43.27 ± 9.97 and 64.20 ± 15.97%, *P* < 0.05) by FA, and Pex19 was tended to be promoted (33.89 ± 16.94%, *P* = 0.06) by FA. However, Pex11a and Pex5 were lower significantly and non-significantly (23.37 ± 0.4%, *P* < 0.05 and 21.22 ± 12.53%, *P* = 0.09, respectively).

### Oxygen Consumption Rate

**Figure 6:**
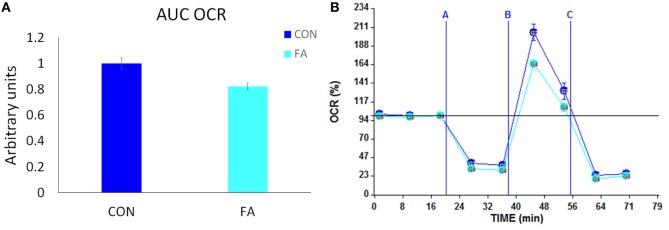
Metabolic profiling of C2C12 cells in in control (CON) and EPA/DHA (FA) medium. **(A)** Total oxygen consumption (reserve capacity) is significantly lower in FA group. Data show the change of oxygen consumption rates (OCR) in FA group compared with CON group. The relative change was calculated in arbitrary units. **(B)** Basal and stimulated mitochondrial OCR in cultured C2C12 cells. OCR traces are expressed as pmol O_2_ per min in C2C12 cells and normalized to cell number. Vertical dashed lines indicate the times of addition of oligomycin (2 µM), FCCP (350 nM), and antimycin A (10 µM). *P < 0.05; *n* = 11.

To confirm whether the metabolism of the cells was affected by FA treatment through changed gene expression related to mitochondria and peroxisome, we measured the oxygen consumption in Seahorse system and calculated the OCR in the two groups normalized to cell number. Results showed that the maximum oxygen consumption capacity stimulated by 350-nM FCCP and inhibited by 10-µM antimycin was significantly decreased (17.79 ± 2.67%, *P* < 0.05) by FA treatment compared with CON group with no FA in the DM (Figure 6).

## Discussion

Long-chain polyunsaturated fatty acids (LCPUFAs) pass through the human placenta and can be delivered to the fetus (17, 29). Moreover, they are present in the breast milk to affect postnatal growth and development (30). Imbalance in PUFA intake, including maternal malnutrition or overnutrition, during fetal development, changes the fatty-acid deposition in the cell membrane as phospholipids and causes implication for cell structure and function (17). Insufficient maternal nutrient intake has been widely studied. The lower level of maternal *n* − 3 fatty acids is related to higher body and abdominal fat mass in childhood (31). On the other hand, maternal obesity, overeating, and overnutrition also increase the risk of metabolic disorders in the fetus and subsequently induce postnatal obesity (32, 33). It was reported that excessive *n* − 3 fatty-acid intake resulted in growth deficiencies such as slower neural transmission times and postnatal growth restriction (34). Animal growth is contributed to bone formation, muscle building, and fat tissue deposition. During later gestation and early postnatal stage, muscle and adipose tissue growth are highly affected by maternal nutrition level. This study tried to discover the effect of *n* − 3 fatty acids on the myoblasts growth in gene expression level. Based on previous studies of EPA and DHA in cell culture study, EPA and DHA are toxic for C2C12 cells when the concentration is >50 µM (35). Therefore, 50 µM was selected in the current study for EPA and DHA as a high concentration without apparent toxicity to C2C12 cells.

During myogenic differentiation, we found that several vital myogenic genes were significantly downregulated in FA-treated group, such as MRF4, MyoD, and MyoG. MRF4, also known as myogenic factor 6 (MYF6), plays a role the in myotube maturation. MyoD and MyoG, as well as MRF4, belong to myogenic regulatory factors, but their functions are slightly different. MyoD is one of the earliest myogenic markers. MyoG is a crucial gene that regulates the secondary muscle fibers maturation (36). Pax 7 is critical for regulating the differentiation of satellite cells (37). Once myoblasts start myogenic differentiation, Pax7 usually does not express. In our study, the Pax7 expression was lower in FA group, although the average decreasing was >60%, the variation of the response is enormous, which indicated the initiation of myogenic differentiation induced by FA was not constant. The results of this study showed that the PUFA treatment inhibits myotube formation and eventually arrests the development of myofibers. These adverse effects were confirmed later in H&E staining by showing fewer and smaller myotubes formed in FA group. However, previous study showed that EPA alone promotes skeletal muscle protein accretion with the L-leucine supplement, but not DHA (38). Leucine is one of the branched-chain amino acids that are highly effective to accelerate skeletal muscle protein synthesis. It may have additional effect on EPA to elevate muscle growth. Our results may indicate that without L-leucine, the effect of EPA on muscle growth might be negative. Also, our data showed that the combination of EPA and DHA might be antagonistic to muscle growth.

Most of the essential genes related to adipogenesis, such as aP2, C/EBPα, C/EBPb, PPARγ, CPT1b, and FAT, were upregulated in FA group. aP2 is also called fatty-acid binding protein 4, for its function of carrying protein for fatty acids to regulate lipid metabolism and mostly expressed in adipocytes. In this study, aP2 gene expression was dramatically stimulated by EPA and DHA treatment, which was 1,700% of the expression in CON group. *n* − 3 fatty acids usually have a high degree of unsaturation, result into the quickly processed fatty-acid oxidation, and stimulate PPARs (39). PPAR isoforms participate in lipid homeostasis and glucose regulation. PPARs target the specific genes, for example, aP2, which contain specific DNA regions called peroxisome proliferator hormone response elements (40). Fatty infiltration in skeletal muscle is accompanying with aging in human (41). These results showed that potentially upregulated adipogenesis in C2C12 cell culture could be triggered by EPA and DHA treatment. Our findings were consisted with an *in vivo* study, which showed upregulated adipogenesis in pig suppled with DHA (42). The upregulated adipogenesis and the decreased myogenic gene expression may indicate that overdosing EPA and DHA is associated with muscle aging. CEBPs are critical for the regulation of genes related to immune and inflammatory responses. However, IL-6 and TNF-a gene expressions are similar between CON and FA group.

As the “powerhouse” of the cell, mitochondria break fuel molecules and provide energy to the cell in the formation of ATP. The mitochondrial oxidative phosphorylation system is considered as the primary source for energy production, which relies on the structure of mitochondrial inner membrane (43). PGC1 family is a group of transcriptional coactivators regulates cellular energy metabolic pathways and mitochondrial biosynthesis (44, 45). ERRα and its down-streaming target gene Mfn2 are key genes that regulate mitochondrial fusion and metabolism (46). TFAM is an essential activator of mitochondrial genome replication. The mRNA expression data of this study showed that PUFA supplement in DM during myogenesis inhibits the expression of the key genes of mitochondrial metabolism and biosynthesis. The decreasing of the mitochondrial biosynthesis was confirmed by measuring the ratio of mtDNA/nDNA. The ratio of mtDNA/nDNA is often used an estimate for the mtDNA copy number per cell (47). It is a high-level indicator if mitochondrial biogenesis as the mitochondrial genome encodes most of the enzymatic subunits of the oxidative phosphorylation system. The decreased mitochondrial metabolism was supported by testing the respiration of the cells, which showed the PUFA-treated cells consumed less oxygen which indicated less-mitochondrial metabolism. Harmed mitochondrial dynamics and biosynthesis negatively affect the regulation of energy expenditure which leads to obesity and diabetes (48), which implies that overdosing EPA and DHA during gestation may have a long-term effect on offspring metabolic disorder.

Peroxisomes are found of playing a significant role in breaking down very long chain fatty acid [greater than C22, such as DHA which is 22:6 (*n* = 3)] through beta-oxidation, but is not coupled to ATP synthesis (49). Peroxisomal membrane proteins (PMPs), which are encoded by peroxisomal biogenesis genes (PEXs), are involved in peroxisome biogenesis (50). Among them, PMP70 and Pex19 are considered as the critical proteins in peroxisomal proliferation and biogenesis (51, 52). The altered expression of PEXs in this study indicated that PUFAs affect peroxisomal biogenesis. The high-potential electrons produced by beta-oxidation in peroxisomes are transferred to yield H2O2, which generates heat and contributes to the accumulation of reactive oxygen species (ROS). The mitochondrial antioxidant system removes ROS production and inhibits ROS production. The lower metabolism of mitochondria induced by EPA and DHA may enhance the oxidative stress in myoblasts differentiation and cause structural and functional muscle abnormalities (53). This may be an explanation of downregulated myogenic gene expression and smaller and fewer myotube formation.

## Conclusion

This study demonstrated the effects of EPA and DHA supplement in C2C12 myogenic differentiation. The key gene expression in myogenesis, adipogenesis, mitochondrial, and peroxisomal biosynthesis in C2C12 cells during myogenic differentiation is affected by EPA and DHA. The myotube formation is inhibited while PUFA treatment upregulates adipogenic gene expression. Strengthened peroxisomal biosynthesis and weakened mitochondrial metabolism and biosynthesis imply that the overdosed PUFAs change energy expenditure, and may cause ROS stress that negatively regulates myogenesis. These indicate that the maternal overdosage of EPA and DHA may influence fetal muscle development, increase intramuscular adipose tissue deposition in offspring, and have a long-term effect on the development of metabolic disease such as obesity and diabetes in adult offspring.

## Author Contributions

YH, T-YH, and JB conceived and designed the experiments. YH and T-YH performed the experiments and analyzed the data. YH wrote the manuscript.

## Conflict of Interest Statement

The authors declare that the research was conducted in the absence of any commercial or financial relationships that could be construed as a potential conflict of interest. The handling editor declared a shared affiliation, though no other collaboration with the authors.

## References

[1] Ruiz-Lopez N Haslam RP Napier JA Sayanova O. Successful high-level accumulation of fish oil omega-3 long-chain polyunsaturated fatty acids in a transgenic oilseed crop. Plant J (2014) 77:198–208. 10.1111/tpj.12378 24308505 PMC4253037

[2] Swanson D Block R Mousa SA. Omega-3 fatty acids EPA and DHA: health benefits throughout life. Adv Nutr (2012) 3:1–7. 10.3945/an.111.000893 22332096 PMC3262608

[3] Janovska P Flachs P Kazdova L Kopecky J. Anti-obesity effect of n-3 polyunsaturated fatty acids in mice fed high-fat diet is independent of cold-induced thermogenesis. Physiol Res (2013) 62:153–61. 23234412 10.33549/physiolres.932464

[4] Buckley JD Howe PR. Anti-obesity effects of long-chain omega-3 polyunsaturated fatty acids. Obes Rev (2009) 10:648–59. 10.1111/j.1467-789X.2009.00584.x 19460115

[5] Hainault I Carolotti M Hajduch E Guichard C Lavau M Fish oil in a high lard diet prevents obesity, hyperlipemia, and adipocyte insulin resistance in rats. Ann N Y Acad Sci (1993) 683:98–101. 10.1111/j.1749-6632.1993.tb35696.x 8352478

[6] Golub N Geba D Mousa SA Williams G Block RC. Greasing the wheels of managing overweight and obesity with omega-3 fatty acids. Med Hypotheses (2011) 77:1114–20. 10.1016/j.mehy.2011.09.016 21981905 PMC3210336

[7] Greenberg JA Bell SJ Ausdal WV. Omega-3 fatty acid supplementation during pregnancy. Rev Obstet Gynecol (2008) 1:162–9. 19173020 PMC2621042

[8] EFSA Panel on Dietetic Products, Nutrition and Allergies (NDA). Scientific opinion on the tolerable upper intake level of eicosapentaenoic acid (EPA), docosahexaenoic acid (DHA) and docosapentaenoic acid (DPA). EFSA J (2012) 10:2815 10.2903/j.efsa.2012.2815

[9] Olsen SF Osterdal ML Salvig JD Mortensen LM Rytter D Secher NJ Fish oil intake compared with olive oil intake in late pregnancy and asthma in the offspring: 16 y of registry-based follow-up from a randomized controlled trial. Am J Clin Nutr (2008) 88:167–75. 10.1093/ajcn/88.1.167 18614738

[10] Lowell BB Shulman GI Mitochondrial dysfunction and type 2 diabetes. Science (2005) 307:384–7. 10.1126/science.1104343 15662004

[11] Stickland NC A quantitative study of muscle development in the bovine foetus (*Bos indicus*). Anat Histol Embryol (1978) 7:193–205. 10.1111/j.1439-0264.1978.tb00795.x 152066

[12] Zhu MJ Ford SP Means WJ Hess BW Nathanielsz PW Du M. Maternal nutrient restriction affects properties of skeletal muscle in offspring. J Physiol (2006) 575:241–50. 10.1113/jphysiol.2006.112110 16763001 PMC1819430

[13] Zhu MJ Han B Tong J Ma C Kimzey JM Underwood KR AMP-activated protein kinase signalling pathways are down regulated and skeletal muscle development impaired in fetuses of obese, over-nourished sheep. J Physiol (2008) 586:2651–64. 10.1113/jphysiol.2007.149633 18372306 PMC2464338

[14] Tong JF Yan X Zhu MJ Ford SP Nathanielsz PW Du M. Maternal obesity downregulates myogenesis and beta-catenin signaling in fetal skeletal muscle. Am J Physiol Endocrinol Metab (2009) 296:E917–24. 10.1152/ajpendo.90924.2008 19176350 PMC2670630

[15] Tong J Zhu MJ Underwood KR Hess BW Ford SP Du M. AMP-activated protein kinase and adipogenesis in sheep fetal skeletal muscle and 3T3-L1 cells. J Anim Sci (2008) 86:1296–305. 10.2527/jas.2007-0794 18344293

[16] Yan X Zhu MJ Xu W Tong JF Ford SP Nathanielsz PW Up-regulation of toll-like receptor 4/nuclear factor-kappa B signaling is associated with enhanced adipogenesis and insulin resistance in fetal skeletal muscle of obese sheep at late gestation. Endocrinology (2010) 151:380–7. 10.1210/en.2009-0849 19887565 PMC2803146

[17] Kabaran S Besler HT. Do fatty acids affect fetal programming? J Health Popul Nutr (2015) 33:14. 10.1186/s41043-015-0018-9 26825664 PMC5025983

[18] Ong ZY Muhlhausler BS Maternal “junk-food” feeding of rat dams alters food choices and development of the mesolimbic reward pathway in the offspring. FASEB J (2011) 25:2167–79. 10.1096/fj.10-178392 21427213 PMC3114523

[19] Anderson EJ Thayne KA Harris M Shaikh SR Darden TM Lark DS Do fish oil omega-3 fatty acids enhance antioxidant capacity and mitochondrial fatty acid oxidation in human atrial myocardium via PPARgamma activation? Antioxid Redox Signal (2014) 21:1156–63. 10.1089/ars.2014.5888 24597798 PMC4142835

[20] Martins AR Nachbar RT Gorjao R Vinolo MA Festuccia WT Lambertucci RH Mechanisms underlying skeletal muscle insulin resistance induced by fatty acids: importance of the mitochondrial function. Lipids Health Dis (2012) 11:30. 10.1186/1476-511X-11-30 22360800 PMC3312873

[21] Flachs P Horakova O Brauner P Rossmeisl M Pecina P Franssen-van Hal N Polyunsaturated fatty acids of marine origin upregulate mitochondrial biogenesis and induce beta-oxidation in white fat. Diabetologia (2005) 48:2365–75. 10.1007/s00125-005-1944-7 16205884

[22] Cavaliere G Trinchese G Bergamo P De Filippo C Raso GM Gifuni G Polyunsaturated fatty acids attenuate diet induced obesity and insulin resistance, modulating mitochondrial respiratory uncoupling in rat skeletal muscle. PLoS One (2016) 11:e0149033. 10.1371/journal.pone.0149033 26901315 PMC4762694

[23] Vaughan RA Garcia-Smith R Bisoffi M Conn CA Trujillo KA. Conjugated linoleic acid or omega 3 fatty acids increase mitochondrial biosynthesis and metabolism in skeletal muscle cells. Lipids Health Dis (2012) 11:142. 10.1186/1476-511X-11-142 23107305 PMC3515476

[24] Jeng JY Lee WH Tsai YH Chen CY Chao SY Hsieh RH. Functional modulation of mitochondria by eicosapentaenoic acid provides protection against ceramide toxicity to C6 glioma cells. J Agric Food Chem (2009) 57:11455–62. 10.1021/jf902021h 19921818

[25] Lee M-S Shin Y Moon S Kim S Kim Y Effects of eicosapentaenoic acid and docosahexaenoic acid on mitochondrial DNA replication and pgc-1α gene expression in c(2)c(12) muscle cells. Prev Nutr Food Sci (2016) 21:317–22. 10.3746/pnf.2016.21.4.317 28078253 PMC5216882

[26] Kuchler RJ Biochemical Methods in Cell Culture and Virology. Stroudsburg: Dowden, Hutchinson & Ross (1977).

[27] Acehan D Vaz F Houtkooper RH James J Moore V Tokunaga C Cardiac and skeletal muscle defects in a mouse model of human Barth syndrome. J Biol Chem (2011) 286:899–908. 10.1074/jbc.M110.171439 21068380 PMC3020775

[28] Nicholls DG Darley-Usmar VM Wu M Jensen PB Rogers GW Ferrick DA. Bioenergetic profile experiment using C2C12 myoblast cells. J Visual Exp (2010) 46:2511. 10.3791/2511 21189469 PMC3159644

[29] Duttaroy AK. Transport of fatty acids across the human placenta: a review. Prog Lipid Res (2009) 48:52–61. 10.1016/j.plipres.2008.11.001 19041341

[30] Jensen RG. Lipids in human milk. Lipids (1999) 34:1243–71. 10.1007/s11745-999-0477-2 10652985

[31] Vidakovic AJ Gishti O Voortman T Felix JF Williams MA Hofman A Maternal plasma PUFA concentrations during pregnancy and childhood adiposity: the Generation R Study. Am J Clin Nutr (2016) 103:1017–25. 10.3945/ajcn.115.112847 26912493 PMC5426536

[32] Vickers MH Breier BH Cutfield WS Hofman PL Gluckman PD. Fetal origins of hyperphagia, obesity, and hypertension and postnatal amplification by hypercaloric nutrition. Am J Physiol Endocrinol Metab (2000) 279:E83–7. 10.1152/ajpendo.2000.279.1.E83 10893326

[33] Dong M Zheng Q Ford SP Nathanielsz PW Ren J. Maternal obesity, lipotoxicity and cardiovascular diseases in offspring. J Mol Cell Cardiol (2013) 55:111–6. 10.1016/j.yjmcc.2012.08.023 22982026

[34] Church MW Jen KL Dowhan LM Adams BR Hotra JW. Excess and deficient omega-3 fatty acid during pregnancy and lactation cause impaired neural transmission in rat pups. Neurotoxicol Teratol (2008) 30:107–17. 10.1016/j.ntt.2007.12.008 18243652

[35] Lee M-S Kim I-H Kim Y Effects of eicosapentaenoic acid and docosahexaenoic acid on uncoupling protein 3 gene expression in c2c12 muscle cells. Nutrients (2013) 5:1660–71. 10.3390/nu5051660 23698161 PMC3708343

[36] Hasty P Bradley A Morris JH Edmondson DG Venuti JM Olson EN Muscle deficiency and neonatal death in mice with a targeted mutation in the myogenin gene. Nature (1993) 364:501–6. 10.1038/364501a0 8393145

[37] von Maltzahn J Jones AE Parks RJ Rudnicki MA. Pax7 is critical for the normal function of satellite cells in adult skeletal muscle. Proc Natl Acad Sci U S A (2013) 110:16474–9. 10.1073/pnas.1307680110 24065826 PMC3799311

[38] Kamolrat T Gray SR. The effect of eicosapentaenoic and docosahexaenoic acid on protein synthesis and breakdown in murine c2c12 myotubes. Biochem Biophys Res Commun (2013) 432:593–8. 10.1016/j.bbrc.2013.02.041 23438435

[39] Grygiel-Gorniak B Peroxisome proliferator-activated receptors and their ligands: nutritional and clinical implications – a review. Nutr J (2014) 13:17 10.1186/1475-2891-13-17 24524207 PMC3943808

[40] Berger J Moller DE. The mechanisms of action of PPARs. Annu Rev Med (2002) 53:409–35. 10.1146/annurev.med.53.082901.104018 11818483

[41] Kirkland JL Tchkonia T Pirtskhalava T Han JR Karagiannides I. Adipogenesis and aging: does aging make fat go MAD? Exp Gerontol (2002) 37:757–67. 10.1016/S0531-5565(02)00014-1 12175476

[42] Huang C-W Chen Y-J Yang J-T Chen C-Y Ajuwon KM Chen S-E Docosahexaenoic acid increases accumulation of adipocyte triacylglycerol through up-regulation of lipogenic gene expression in pigs. Lipids Health Dis (2017) 16:33. 10.1186/s12944-017-0428-3 28173868 PMC5297193

[43] Hüttemann M Lee I Pecinova A Pecina P Przyklenk K Doan JW. Regulation of oxidative phosphorylation, the mitochondrial membrane potential, and their role in human disease. J Bioenerg Biomembr (2008) 40:445. 10.1007/s10863-008-9169-3 18843528

[44] Finck BN Kelly DP. PGC-1 coactivators: inducible regulators of energy metabolism in health and disease. J Clin Invest (2006) 116:615–22. 10.1172/JCI27794 16511594 PMC1386111

[45] Scarpulla RC. Metabolic control of mitochondrial biogenesis through the PGC-1 family regulatory network. Biochim Biophys Acta (2011) 1813:1269–78. 10.1016/j.bbamcr.2010.09.019 20933024 PMC3035754

[46] Soriano FX Liesa M Bach D Chan DC Palacin M Zorzano A. Evidence for a mitochondrial regulatory pathway defined by peroxisome proliferator-activated receptor-gamma coactivator-1 alpha, estrogen-related receptor-alpha, and mitofusin 2. Diabetes (2006) 55:1783–91. 10.2337/db05-0509 16731843

[47] Phillips NR Sprouse ML Roby RK. Simultaneous quantification of mitochondrial DNA copy number and deletion ratio: a multiplex real-time PCR assay. Sci Rep (2014) 4:3887. 10.1038/srep03887 24463429 PMC4894387

[48] Liesa M Shirihai OS. Mitochondrial dynamics in the regulation of nutrient utilization and energy expenditure. Cell Metab (2013) 17:491–506. 10.1016/j.cmet.2013.03.002 23562075 PMC5967396

[49] Singh I. Biochemistry of peroxisomes in health and disease. Mol Cell Biochem (1997) 167:1–29. 10.1023/A:1006812302485 9059978

[50] Smith JJ Aitchison JD. Peroxisomes take shape. Nat Rev Mol Cell Biol (2013) 14:803–17. 10.1038/nrm3700 24263361 PMC4060825

[51] Schrader M Bonekamp NA Islinger M. Fission and proliferation of peroxisomes. Biochim Biophys Acta (2012) 1822:1343–57. 10.1016/j.bbadis.2011.12.014 22240198

[52] Imanaka T Aihara K Suzuki Y Yokota S Osumi T The 70-kDa peroxisomal membrane protein (PMP70), an ATP-binding cassette transporter. Cell Biochem Biophys (2000) 32:131–8. 10.1385/CBB:32:1-3:131 11330039

[53] Couillard A Prefaut C. From muscle disuse to myopathy in COPD: potential contribution of oxidative stress. Eur Respir J (2005) 26:703–19. 10.1183/09031936.05.00139904 16204604

